# Multilocus Intron Trees Reveal Extensive Male-Biased Homogenization of Ancient Populations of Chamois (*Rupicapra* spp.) across Europe during Late Pleistocene

**DOI:** 10.1371/journal.pone.0170392

**Published:** 2017-02-01

**Authors:** Trinidad Pérez, Margarita Fernández, Sabine E. Hammer, Ana Domínguez

**Affiliations:** 1 Departamento de Biología Funcional, Universidad de Oviedo, Julián Clavería 6, Oviedo, Spain; 2 Department of Pathobiology, Institute of Immunology, University of Veterinary Medicine Vienna, Veterinaerplatz 1, Vienna, Austria; Smithsonian Conservation Biology Institute, UNITED STATES

## Abstract

The inferred phylogenetic relationships between organisms often depend on the molecular marker studied due to the diverse evolutionary mode and unlike evolutionary histories of different parts of the genome. Previous studies have shown conflicting patterns of differentiation of mtDNA and several nuclear markers in chamois (genus *Rupicapra*) that indicate a complex evolutionary picture. Chamois are mountain caprine that inhabit most of the medium to high altitude mountain ranges of southern Eurasia. The most accepted taxonomical classification considers two species, *R*. *pyrenaica* (with the subspecies *parva*, *pyrenaica* and *ornata*) from southwestern Europe and *R*. *rupicapra* (with the subspecies *cartusiana*, *rupicapra*, *tatrica*, *carpatica*, *balcanica*, *asiatica* and *caucasica*) from northeastern Europe. Phylogenies of mtDNA revealed three very old clades (from the early Pleistocene, 1.9 Mya) with a clear geographical signal. Here we analyze a set of 23 autosomal introns, comprising 15,411 nucleotides, in 14 individuals covering the 10 chamois subspecies. Introns offered an evolutionary scenario that contrasts with mtDNA. The nucleotidic diversity was 0.0013± 0.0002, at the low range of what is found in other mammals even if a single species is considered. A coalescent multilocus analysis with *BEAST indicated that introns diversified 88 Kya, in the late Pleistocene, and the effective population size at the root was lower than 10,000 individuals. The dispersal of some few migrant males should have rapidly spread trough the populations of chamois, given the homogeneity of intron sequences. The striking differences between mitochondrial and nuclear markers can be attributed to strong female philopatry and extensive male dispersal. Our results highlight the need of analyzing multiple and varied genome components to capture the complex evolutionary history of organisms.

## Introduction

Phylogenetic studies try to reconstruct the evolutionary history of organisms from the comparison of parts of the genome. DNA sequences are compared and the relationships among species or groups are usually represented as dichotomous trees with branches that never reconnect. After the great development of molecular analyses in the last few decades it was frequently observed that the trees obtained for different markers were discordant. In some cases, these observations can be interpreted as incomplete lineage sorting, and coalescent methods have been implemented for species assignment from the integration of gene trees [1]. But the alternative explanation in terms of reticulate evolution, where populations that diverge in isolation eventually reconnect and interchange parts of genome is likely to be true [2]. In the last decade, examples of interchange between animal lineages and its role in posterior differentiation are growing exponentially [3, 4] and the paradigm of the “web-of-life metaphor” [5] has received growing interest. As in other bovids, the patterns of differentiation for different molecular markers in chamois (*Rupicapra* spp.) can be best explained as reticulate evolution [2].

**Fig 1:**
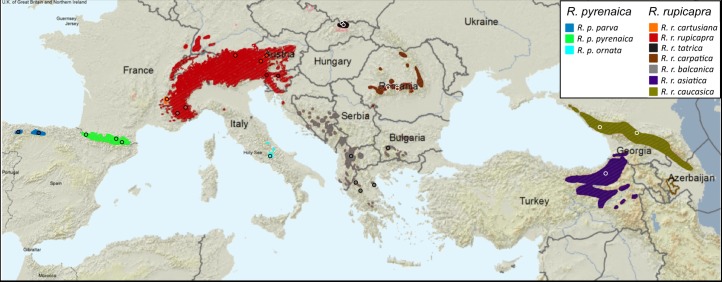
Geographic distribution of the subspecies of the genus *Rupicapra* [19]. The map was modified from the distribution map on the IUCN Red List.

Chamois are mountain caprine that inhabit most of the medium to high altitude mountain ranges of southern Eurasia (Fig 1). At present, the most accepted classification of chamois considers two species, *R*. *pyrenaica* and *R*. *rupicapra*, [6, 7]: *Rupicapra pyrenaica* (with the subspecies *parva*, *pyrenaica* and *ornata*) from southwestern Europe, and *R*. *rupicapra* (with the subspecies *cartusiana*, *rupicapra*, *tatrica*, *carpatica*, *balcanica*, *asiatica* and *caucasica*) from northeastern Europe. However, the taxonomy of the genus has been subject to continuous revisions since the beginning of the twentieth century. In 1914, Camerano [8] distinguished the species *R*. *ornata* on the basis of skull and horn morphometrics, a viewpoint that was also expressed by Cabrera [9]. Subsequently, Couturier [10] considered the ten populations of chamois as a single species, but later work based on skull evaluations [11], electrophoretic data [12] and different coat pattern, as well as several courtship behaviour patterns [13], suggested the treatment as two species. Analysis of genetic variation in a limited number of subspecies for minisatellites [14] and RFLPs of mitochondrial DNA [15] provided some support to this classification. Just a few years ago, new taxonomical classifications of the genus have been proposed. Valdez [16] proposed the recognition of six species and Groves and Grubb [17] increased the number of species up to seven. These new classifications are both based on morphological traits and geographical distribution. The mitochondrial phylogeny, that is frequently used to diagnose species, showed three main lineages, all originated in a close period [18–20], instead of two lineages, one for each of the nominal species. These three mtDNA clades were referred to as W, C and E after its restricted geographic distribution in either west, central or east Eurasia [18]. Nuclear markers as microsatellites and the melacortin-1 receptor gene (MC1R) formed three clearly defined groups as well; however, those groups did not exactly matched the mitochondrial lineages but differed in the boundaries at central Europe [19, 21–23]. The populations in the Iberian Peninsula (*R*. *pyrenaica parva* and *R*. *pyrenaica pyrenaica*) grouped into clade W, the population in the Apennines (*R*. *pyrenaica ornata*) was of clade C and the populations to the East of the Alps were of clade E, both for mitochondrial and nuclear loci. Only the *R*. *rupicapra* populations in the west Alps showed discordance among loci: the small population in the Massif of Chartreuse (*R*. *rupicapra cartusiana*) was of mitochondrial type C and of nuclear type E and several individuals in the west Alps were of mtDNA type W and nuclear type E. The partial discordances between markers indicated events of range overlap and hybridization among highly divergent lineages in the central area of the distribution, with a major effect of the Alpine barrier on west-east differentiation [19].

With regard to the timing of differentiation of *Rupicapra*, the divergence between the main mtDNA clades has been estimated around 1.9 mya [19, 24, 25], at the Early Pleistocene following the recent “Formal Subdivisions of the Pleistocene Series/Epoch” of the subcommission on Quaternary Stratigraphy [26] that we will adopt along the paper. This is by far older than the age of the most ancient *Rupicapra* fossils in Europe that were discovered in the Balkans and correspond to the beginning of the middle Pleistocene, between 780 and 750 Kya [27]. The molecular dating using microsatellites has given much more recent separation times [21] and, to integrate mtDNA and microsatellite information, this was interpreted as a result of saturation in this kind of marker [19]. But it is remarkable that the study of chromosome Y, based on both microsatellites and nucleotide substitutions, showed a striking homogeneity among all chamois populations compatible with a very young origin of the male lineage [23]. Nevertheless, as discussed in the referred paper, the specific characteristics of the Y-chromosome regarding recombination and selection can be invoked to explain its low diversity among populations.

To study the causes of the incongruences between the mtDNA and the nuclear markers analysed so far it is convenient to study the nucleotidic variation of multiple neutral markers and investigate their phylogenies and evolutionary histories both through the traditional methods of concatenation and by multilocus coalescent methods that incorporate lineage sorting to explain difference among phylogenies. These kind of studies provided essential information about the evolutionary histories of other mammals [28, 29].

Here we analyze multiple independently inherited autosomal introns to examine whether there are discrepancies among them and/or incongruences with other phylogenies based on mtDNA, the Y chromosome or the MC1R gene. We sequenced 23 independently inherited autosomal introns in 14 individuals from the ten populations considered different subspecies of *Rupicapra*. There were 15,411 nucleotides in total for the 23 introns. The diversity and phylogenetic relationships among subspecies were investigated through methods of AMOVA (Analysis of Molecular Variance) and Bayesian clustering as well as phylogenetic networks and trees. The congruence among phylogenies obtained from different introns was studied by coalescent-based multilocus phylogenetic approaches [30]. We study whether different introns present conflicting phylogenies and compare these results with previous studies on mtDNA, other nuclear biparental markers and the chromosome Y to explore the role of different components of the genome in the differentiation and evolutionary history of chamois.

## Materials and Methods

### Samples

Fourteen individuals covering the ten subspecies of the genus *Rupicapra*, and already studied for other markers, were analysed (see S1 Table): *Rupicapra pyrenaica parva* (samples CBWo04 and CBEo12), *Rupicapra pyrenaica pyrenaica* (samples PYWo15 and PYEo13), *Rupicapra pyrenaica ornata* (sample ANo01), *Rupicapra rupicapra cartusiana* (samples CHAv01 and CHAv04), *Rupicapra rupicapra rupicapra* (samples ALWo09 and ALEo03), *Rupicapra rupicapra tatrica* (sample Tao02), *Rupicapra rupicapra carpatica* (sample CPo03), *Rupicapra rupicapra balcanica* (sample BAo16), *Rupicapra rupicapra asiatica* (sample TUo01) and *Rupicapra rupicapra caucasica* (sample CUo05).

### Intron selection, amplification and sequencing

Introns for this study were selected among the previously designed for the study of mammal phylogenies [29, 31, 32]. We selected a set of 30 introns, unlinked in the genomes of cattle (*Bos taurus*) and sheep (*Ovis aries*). In some cases the given primer sequences were modified according to the reference sequence in these species (Btau 4.6.1 and Oar 3.1) to improve specificity (S2 Table). Seven introns were discarded due to poor amplification and 23 introns were finally studied. Following are the genes and the intron number in parentheses: TRAPPC10 (9), CLCA1 (12), LRGUK (14), SEL1L3 (20), COPE (6), ABCA1 (49), HDAC2 (13), PABPN1 (2), SPTBN1 (31), ATP12A (14), GAD2 (1), AZIN1 (8), LYVE1 (5), PTGS2 (3), FGB (8), GGA3 (4), PNN (1), SCN5A (26), RIOK3 (6), CARHSP1 (2), TUFM (9), ZFYVE27 (6), KLC2 (11).

Amplifications were done in a final volume of 20 μl containing 2 μl with about 50 ng DNA, 0.5 μM of each primer, 1x PCR buffer, 2.5–5.0 mM MgCl_2_, 250 μM of each dNTP and 1U of Biotools DNA Polymerase (B & M Laboratories, Madrid, Spain). Amplification was carried out in PE GeneAmp PCR 9700 thermal cycler (Applied Biosystems, Foster City, CA) with an initial step of 5 min at 95°C, 38 cycles of 30 s at 95°C, 30 s at the appropriate annealing temperature for each primer pair (see S2 Table) and 40 s at 72°C, followed by 10 min at 72°C. PCR products were electrophoresed along with size standards in 2% agarose gel in 1x Tris–borate–EDTA and visualized by UV. The PCR-amplified products were purified with the illustra™ ExoStar™ 1-Step (GE Healthcare). Both strands of PCR products were sequenced with PCR primers and the BigDye Terminator v3.1 Cycle Sequencing Kit (Applied Biosystems). Sequencing products were purified with isopropanol precipitation and sequenced in an ABI 310 Genetic Analyzer (Applied Biosystems).

### Sequence alignment

The sequence data were analyzed and assembled using Sequencher 4.9 (Gene Codes Corp., Ann Arbor, MI) and manually checked and edited (GenBank Acc. Nos in S3 Table). Heterozygous insertions/deletions (indels) were resolved with the aid of Indelligent [33]. Then, we inferred haplotypes by reading the FASTA files, including ambiguity codes to represent heterozygous sites, into DnaSP 5 [34] and using the algorithm implemented in Phase 2.1 [35] inside DnaSP. Haplotypes were reconstructed allowing recombination and with the default options in Markov Chain Monte Carlo (MCMC) and a cut off of 95%.

### Sequence diversity and population structure

Haplotypic and nucleotidic diversities were computed in DnaSP 5 [34] for the complete dataset and for *R*. *pyrenaica* and *R*. *rupicapra*, separately. A matrix of genotypes coded from the haplotypes deduced by Phase was used to define the most likely number of clusters of individuals. The software STRUCTURE 2.3.4 [36] was used to define clusters and assign individuals into them. Simulations were based in the admixture model with correlated allele frequencies and without *a priori* information about population of origin. We used different values of K, from one to five. For each K tested, we ran STRUCTURE 20 times for 200,000 steps, after a burn-in period of 50,000 steps. The most likely value of K was estimated following Evano et al. (2005) [37]. The programme also calculates the fractional membership of each individual in each cluster (Q) and provides the corresponding plots. In addition, correspondence among haplotype genotypes was studied by the Principal Coordinate Analysis (PCoA) implemented in Genalex 6.5 [38]. Further, PCoA of nucleotidic variation was performed from a matrix of individual distances obtained in DnaSP following the model of Jukes Cantor. The matrix of distances was imported in Genalex where the PCoA was carried out.

Differences among the obtained clusters were further evaluated with Arlequin 3.5 [39] after importing the data from DnaSP. AMOVA among groups defined by PCoA was done both at the haplotypic and the nucleotidic level to obtain pairwise Fst and PhiST values and their significance.

The evolutionary relationships between haplotypes of the different introns were analysed by a Median Joining Network [40] constructed with NETWORK 4.6.1.2 (Fluxus Technology Ltd.). The parameter ε was set to zero (default) to obtain a sparse spanning network. Gaps were treated as a fifth character state.

### Phylogenetic analysis

We constructed phylogenies from the datasets of 14 individuals representing the two species of the genus *Rupicapra* by two kinds of approaches. At first, we employed a species tree method that uses coalescence to incorporate gene tree heterogeneity due to incomplete lineage sorting into one species phylogeny. Secondly, we used concatenation methods including the sequences of *Ovis aries* and *Bos taurus* as outgroups. Finally, we compared the phylogenetic signal obtained with introns to the phylogenies obtained with other nuclear sequences (the SRY promoter and the MC1R gene) and with mitochondrial sequences.

#### Analysis of Introns by concatenation methods

Phylogenetic analyses using Neighbour-Joining (NJ), Maximum-Likelihood (ML) or Bayesian approaches were used to construct phylogenetic trees from the concatenated sequence of 23 introns, after excluding heterozygous and indel positions (15,382 base pairs in length). The NJ tree based on Jukes-Cantor distance was constructed with MEGA 6. The topology of the tree was further investigated by ML with the Heuristic Method of the Nearest-Neighbor-Interchange. Bayesian analysis was conducted using the MCMC method implemented in BEAST 2.1 [41]. We used a strict clock and a Yule speciation process as priors. The model of nucleotide substitution was the Hasegawa-Kishino-Yano (HKY) with the empirical base frequencies, as determined in MEGA. The reliability of the nodes was assessed by 1,000 bootstrap replicates [42] under NJ and ML and by the posterior probability of the nodes under the Bayesian approach (BPP). Divergence times were estimated with BEAST, using two calibration points based on the fossil record and using soft bounds to account for uncertainty [43]. We used the ages and prior probability distributions given in Bibi [44]. The two calibration points were crown Bovidae with a normal prior (mean = 18 Ma, standard deviation = 1 Ma), based on *Eotragus noyei*; and crown Caprini with a normal prior (mean = 8.9 Ma, standard deviation = 2 Ma) based on *Aragoral mudejar* (see Additional File 1 in Bibi 2013). No monophyletic constraints were used. All the analyses were run for 10^9^ generations with tree and parameter sampling every 100,000 generations. A burn-in of 10% was used and the convergence of all parameters assessed using the software TRACER 1.6 [45]. The Maximum clade credibility criterion tree was obtained with TreeAnnotator 2.1 with a posterior probability limit of 50%. Trees obtained by the different methods were visualized with FigTree 1.4 [46].

#### Multilocus coalescent analysis of intron sequences

We performed a multilocus coalescent analysis in *BEAST 2.1, that coestimates multiple gene trees embedded in a shared species tree along with the effective population size of both extant and ancestral species [30, 41]. For the coalescent analysis we used the 23 intron dataset with 28 sequences each, incorporating intra-individual variation. The same substitution model and clock model were used for different loci, while the tree models were unlinked across loci (after the lack of convergence of the MCMC when the substitution models and/or the clock models were unlinked across loci). Model of sequence evolution was HKY with substitution rate equal to 1 and empirical nucleotide frequencies. We used a strict clock with the clock rate set to 1. We used a Yule prior on the species tree and the piecewise linear with constant root on the population size model. Two replicates were run for 10^9^ generations with tree and parameter sampling each 100,000 generations. After checking the convergence of the two replicates in TRACER we fixed a burn-in of 30% that led to a ESS>>200 (= 844) for the three eight. The two runs were combined in LogCombiner 2.1 and DensiTree 2.0 [47] was used to generate a cloudogram of the distribution of species trees. We used the substitution rate of 3.5x10^-9^ substitutions per site per year given for artiodactyl lineage [48] for divergence time estimates. The effective size (Ne) of the branches in the tree was obtained, following the instructions given in the *BEAST tutorial 2.0.3 [49], from the dmv1 and dmv2 values in the summary tree using the above substitution rate and a generation time of 6.24 years [50].

### Comparing nuclear and mitochondrial datasets

We evaluated the phylogenetic signal obtained from introns by comparison with other sets of data previously obtained: two sets of nuclear data, Y-chromosomal data (a fragment of 531 nt of the SRY promoter) [23] and the melacortin-1 receptor gene (MC1R, 954 nt) [22]} and a mitochondrial data set consisting of sequences of five mtDNA regions (CYTB, ND1, 12S, tRNApro and Control Region, 1646 nt) [18, 19]. The GenBank Acc. Numbers of the sequences are given in the S4 Table.

Coalescent analysis of intron sequences together with other nuclear sequences, the SRY promoter and the MC1R gene, were performed in *BEAST 2.4.3. Substitution models and clock models were unlinked for introns, SRY promoter, and the MC1R gene. Tree models were unlinked across loci. Model of sequence evolution was HKY with substitution rate equal to 1 and empirical nucleotide frequencies. We used a strict clock with the clock rate set to 1 for introns, so that the rates of the other two partitions are estimated in relation to introns. We used a Yule prior on the species tree and the Piecewise linear with constant root on the population size model. Two replicates were run for 1,000 million generations with tree and parameter sampling each 100,000 generations, as before. A burn-in of 10% was used and the convergence of all parameters checked using the software TRACER. The Maximum clade credibility criterion tree was obtained with TreeAnnotator, using a burn-in of 10,000 trees and with mean node heights. Trees were visualized with FigTree 1.4.

In addition, we performed the *BEAST analysis including also mitochondrial sequences (CYTB, ND1, 12S, tRNApro and Control Region, 1646 nt). We used the same parameters as before, with substitution and clock models unlinked for the four data sets. Two replicates were run for 2,000 million generations with tree and parameter sampling each 200,000 generations. A burn-in of 10% was used and the convergence of all parameters checked using the software TRACER.

After the lack of convergence of the MCMC when checked in TRACER, we performed the comparison of nuclear and mtDNA topologies in a coalescent framework. We analysed nuclear sequences in *BEAST using the three main clusters defined by mtDNA as prior for the species tree. The posterior probabilities of the two topologies were compared by the Bayes Factor that quantifies the evidence provided by the data in favour of one hypothesis as opposed to another [51]. The marginal likelihoods of the models with or without prior were calculated in TRACER using the smoothed harmonic mean estimator and their standard errors were estimated from 1000 bootstrap replicates [52, 53]. Differences between log marginal likelihoods correspond to log Bayes factors. A log Bayes factor of 0 implies that the two models under comparison are equally likely, while values greater than 2 (Bayes Factor>100) represent very strong evidence in favour of the model with higher likelihood [51]

## Results

### Basic variability analysis and population structure

We sequenced and analysed 15,411 nucleotides from 23 independent nuclear loci in 14 chamois including representatives of the 10 recognised subspecies. In the 15,411 bp of intronic sequences, we found only 67 variable sites (60 excluding indels), which gives a proportion of 0.0043 polymorphic sites per nucleotide (Table 1). We found a total of 75 haplotypes at the 23 intron loci, the number of haplotypes per locus varied between 1 and 7 with a mean of 3.26. The overall haplotypic diversity was 0.42 and the overall nucleotide diversity was 1.27 differences per 1000 sites.

**Table 1:** Genetic diversity in chamois. Estimates are provided for each putative especies, *R*. *pyrenaica* (Rpyr) and *R*. *rupicapra* (Rrup), separately and for the total.

Intron	Alignement length	S Rpyr	S Rrup	S Total	N° ht Rpyr	N° ht Rrup	N° ht Total	Hd Rpyr	Hd Rrup	Hd total	π Rpyr	π Rrup	π total
**ABCA1(49)**	627	0	1	1	1	2	2	0	0.50±0.06	0.39±0.08	0	0.80±0.10	0.62±0.13
**ATP12A(14)**	660	1	1	2	2	1	3	0.36±0.16	0.21±0.12	0.56±0.06	0.54±0.24	0.32±0.18	0.98±0.15
**AZIN1(8)**	568	2	3	5	2	4	6	0.36±0.16	0.74±0.06	0.82±0.03	1.25±0.56	1.77±0.25	2.95±0.23
**CARHSP1(2)**	660	1	1	2	2	2	3	0.36±0.16	0.21±0.12	0.26±0.10	0.54±0.24	0.32±0.18	0.42±0.17
**CLCA1(11)**	847	1	0	1	2	1	2	0.36±0.16	0	0.42±0.08	0.42±0.19	0	0.50±0.09
**COPE(6)**	1024	3	2	4	3	4	7	0.69±0.10	0.48±0.13	0.75±0.07	1.15±0.30	0.64±0.19	1.26±0.15
**FGB(8)**	580	2	0	2	3	1	3	0.62±0.14	0	0.45±0.09	1.23±0.34	0	0.97±0.23
**GAD2-1(1)**	665	2	1	3	3	2	4	0.51±0.16	0.42±0.09	0.67±0.06	1.24±0.41	0.64±0.15	1.25±0.18
**GGA3(4)**	611	1	0	1	2	1	2	0.36±0.16	0	0.14±0.08	0.58±0.26	0	0.23±0.14
**HDAC2(13)**	658	1	0	4	2	1	3	0.56±0.07	0	0.54±0.06	0.84±0.11	0	2.66±0.37
**KLC2(11)**	418	1	3	4	2	3	5	0.47±0.13	0.22±0.12	0.62±0.08	1.12±0.32	1.03±0.68	3.17±0.41
**LRGUK(14)**	713	0	0	0	1	1	1	0	0	0	0	0	0
**LYVE1(5)**	538	2	0	2	3	1	3	0.69±0.10	0	0.32±0.11	1.53±0.33	0	0.62±0.22
**PABPN1(2)**	704	2	0	2	2	1	2	0.36±0.16	0	0.14±0.08	1.01±0.45	0	0.39±0.24
**PNN(1)**	654	1	3	5	2	5	7	0.36±0.16	0.78±0.06	0.84±0.04	0.54±0.24	2.32±0.16	2.77±0.19
**PTGS2(3)**	685	1	0	1	2	1	2	0.36±0.16	0	0.42±0.08	0.52±0.23	0	0.62±0.11
**RIOK3(6)**	657	2	0	2	2	1	2	0.36±0.16	0	0.42±0.08	1.08±0.48	0	1.29±0.23
**SCN5A(26)**	647	1	4	7	2	3	5	0.36±0.16	0.39±0.13	0.68±0.07	0.55±0.25	1.78±0.64	3.71±0.35
**SEL1L3(20)**	830	0	1	1	1	2	2	0	0.37±0.11	0.25±0.09	0	0.44±0.14	0.31±0.11
**SPTBN1(31)**	681	0	0	0	1	1	1	0	0	0	0	0	0
**TRAPPC10(9)**	558	3	1	6	4	2	6	0.82±0.07	0.21±0.12	0.66±0.09	2.43±0.40	0.37±0.21	3.18±0.48
**TUFM(9)**	756	0	0	0	1	1	1	0	0	0	0	0	0
**ZFYVE27(6)**	670	5	0	5	3	1	3	0.51±0.16	0	0.20±0.10	3.32±0.97	0	1.39±0.66
**TOTAL**	15411	32	21	60	48	42	75	0.37±0.05	0.20±0.05	0.41±0.05	0.86±0.17	0.45±0.14	1.27±0.24

Intron: gene name and intron number in the genome of *Bos Taurus*.

S: number of polymorphic sites.

N° ht: number of haplotypes.

Hd: haplotypic diversity ± Standard deviation.

π: nucleotide diversity ± Standard deviation. Values have been multiplied by 1000.

**Fig 2:**
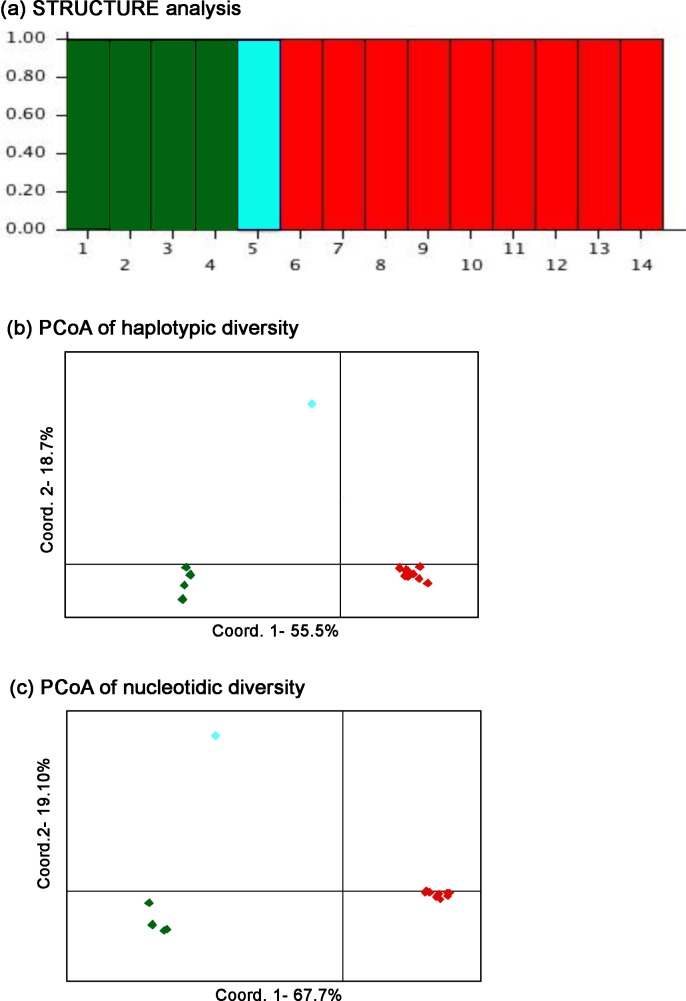
Analysis of population structure. Genetic clusters obtained with the software STRUCTURE (a), PCoA of haplotypic diversity (b) and PCoA of nucleotidic diversity (c). The chamois from Iberia (*parva* and *pyrenaica*) are represented in green, the population from the Apennines (*ornata*) is represented in light blue and the rupicapra cluster, the east populations (*cartusiana*, *rupicapra*, *tatrica*, *carpatica*, *balcanica*, *asiatica*, *caucasica*), are in red.

**Fig 3:**
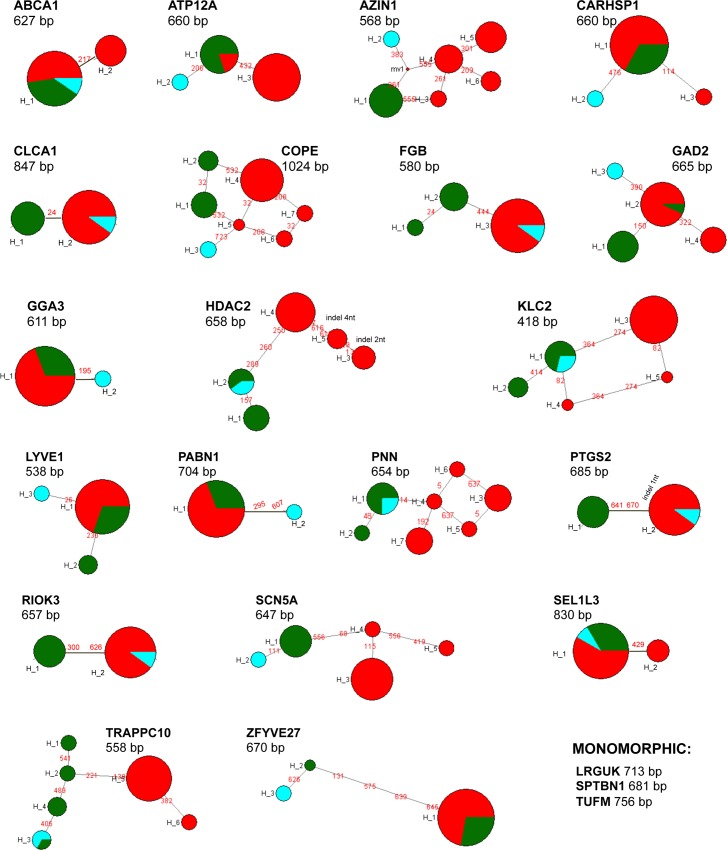
Median-Joining networks of variation at 20 intron loci (other 3 were monomorphic), in chamois. The size of pie areas corresponds to haplotypic frequencies and the proportion accounted for by the chamois from Iberia (*parva* and *pyrenaica*), the Apennines (*ornata*), and the east populations (*cartusiana*, *rupicapra*, *tatrica*, *carpatica*, *balcanica*, *asiatica*, *caucasica*) are represented in green, blue and red, respectively. Inferred mutations in each are indicated and the indels marked with an arrowhead. Intron names and the corresponding alignment lengths are shown above each network.

The analysis with the software STRUCTURE revealed three clusters (K = 3 gives the maximum likelihood value) (Fig 2). The three clusters correspond to the individuals from the Iberian Peninsula (subspecies *R*. *pyrenaica parva* and *R*. *pyrenaica pyrenaica*), the single individual from the Apennines (subspecies *R*. *pyrenaica ornata*) and the chamois populations from the Alps, including the Massif of Chartreuse, to the Caucasus (the seven subspecies of the species *R*. *rupicapra*). From now on, we will refer to these three clusters as iberica, ornata and rupicapra, respectively. The three clusters are clearly visualized in the PCoA of haplotypic diversity, with the two first principal components accounting for 74% of the variation, and in the PCoA analysis of nucleotide diversity (Fig 2), where the first two axis account for 87% of variation. The amount of variation between groups relative to the variation within group, both at the haplotypic and the nucleotidic level (Fst and PhiST values, respectively), is large and significant. The values of Fst are between 0.63 and 0.78 and those of Phi ST between 0.78 and 0.82 (S5 Table). However, mean pairwise differences per nucleotide between these three clusters are low (Table 2), in the order of 0.1–0.2%. Intron variability is very low with three loci monomorphic, other seven with only two haplotypes and the other showing short spanning networks (Fig 3). Haplotypes were shared between the nominal species *R*. *pyrenaica* and *R*. *rupicapra* for 16 of the 23 introns analysed. Only 3 out of the 23 introns showed haplotypes not shared among the three identified clusters.

**Table 2:** Mean pairwise nucleotide differences using the correction of Jukes-Cantor, among *Rupicapra* population groups.

Group	iberica	ornata	rupicapra
**iberica**	0.36	1.71	1.84
**ornata**	1.53	0.00	2.26
**rupicapra**	1.45	2.04	0.43

Values have been multiplied by 1000.

Above diagonal: Differences between populations (PiXY).

Diagonal elements: Differences within population (PiX).

Below diagonal: Corrected average pairwise difference (PiXY-(PiX+PiY)/2).

### Phylogenetic analysis of intron sequences

**Fig 4:**
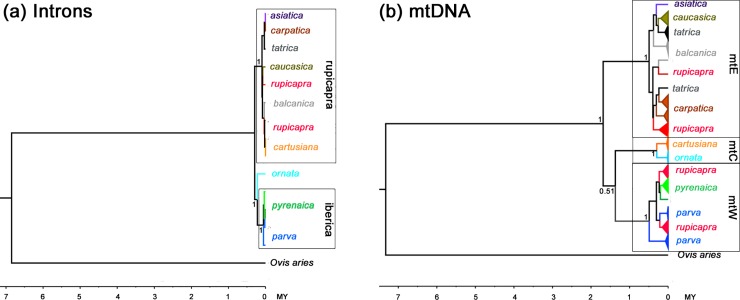
Comparison of intron and mtDNA gene trees. Standard BEAST analysis of: a) concatenated intron sequences (15382 nt) and b) concatenate mtDNA sequences (1646 nt), modified from [19]. The numbers at the nodes are posterior probabilities.

We analysed the sequences of the 23 introns both trough the concatenation of the sequences of the different loci and through multilocus coalescence. First, we did the phylogenetic analysis on the concatenated data. The sequences of the 23 introns in each of the 14 individual chamois and the corresponding sequences of *Ovis aries* and *Bos taurus* from the GenBank were concatenated. Heterozygote positions were removed resulting in a matrix of 16 specimens X 15 382 sites. The topologies of the trees obtained by NJ, ML and Bayesian methods are different to some extent in the position of *ornata* that groups with *rupicapra* in the NJ tree, although with a low bootstrap support of 0.53, but in the ML and Bayesian trees *ornata* groups with the clades from Iberia even though also with low support, 0.54 and 0.49, respectively. The topology and divergence for intron sequences contrasts with that obtained for mtDNA in a previous work [19]. The trees obtained for the two markers with standard BEAST analysis can be seen in Fig 4. The root height for *Rupicapra* introns is 0.0008 substitutions per nucleotide against a root of 0.0378 for mtDNA. The age of root of genus *Rupicapra* estimated from the Bayesian analysis of the concatenated intron sequences was 292 ky (95% HDP 241–527 ky) to be compared with 1.93 mya (1.56–2.33 CI, 95%HPD).

**Fig 5:**
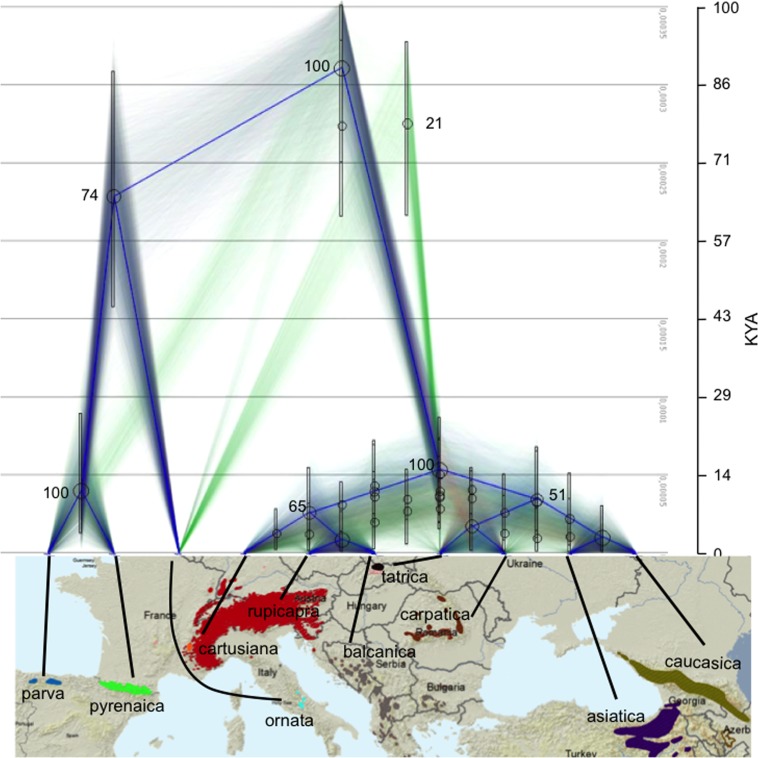
Cloudogram of species trees from *BEAST analysis based on 23 autosomal introns. The consensus tree is superimposed in blue. Dots at nodes indicate posterior support that is also indicated for major nodes. Node bars correspond to 95% credibility interval (95% HDP) times of divergence.

The phylogenetic signal of independent loci can be also studied by a multilocus coalescence approach using *BEAST. This program uses chain Monte Carlo algorithms for Bayesian inference of species trees from multiple gene trees. The multilocus analysis yielded a topology with three main clades corresponding to the previously defined clusters iberica, ornata and rupicapra (Fig 5). There is uncertainty in the relationships of these three clades: ornata is placed as sister of iberica with 74% support and with rupicapra with 21% support indicating the conflict among evolutionary signals among loci. Heights of alternative nodes connecting these three clades show overlapping 95% HDP limits denoting a contemporaneous differentiation. A clade consisting of the two populations of the Iberian Peninsula (*parva* and *pyrenaica*) is recovered with 100% statistical support. All populations of Eastern Europe (*cartusiana*, *rupicapra*, *tatrica*, *carpatica*, *balcanica*, *asiatica*, *caucasica*) form other group, also with high statistical support. Attending to relationships within rupicapra, pairs of populations geographically close are placed as sister groups in the cloudogram, but with moderate posterior probabilities, and alternative topologies are also supported. Divergence between the three main clades was placed between 43,000 and 101,000 years (Table 3 and Fig 5). Subsequently, the two populations of the Iberian Peninsula in one side and the populations occupying the Alps, the East of Europe and the Caucasus in the other diverged more than 10,000 years ago during the Würm Period in the late Pleistocene. The differentiation between neighbour populations should have occurred during the late Holocene, the confidence limits of the 95% HDP reach current time. The coalescent analysis also includes population effective size in the different nodes as a factor of differentiation (Table 3). Effective population size for the ancestral population is low, consistent with the general small diversity in the entire genus.

**Table 3:** Node ages and effective population sizes (Ne).

Node	Years	95% HDP		Ne	95% HDP	
**Root**	88493	71238	101092	7694	4356	11321
**R. pyrenaica**	65295	42939	89325	4049	1378	7140
**R. rupicapra**	15104	1417	23779	3969	1645	6687
**pyr-parva**	11568	7317	23869	3259	1086	5779
**(rup-bal)-cat**	7390	1325	14587	3881	869	7525
**(tat-cap)-(cau-asi)**	9619	2799	17135	2749	378	5657
**rup-bal**	6447	0	6447	3752	754	7375
**tat-cap**	4750	1	11551	2251	196	5047
**asi-cau**	267	1	72	3227	579	6685

The values were obtained from *BEAST assuming a substitution rate of 3.5x10E-9 substitutions/site/year [47] and a generation time of 6.24 years/generation [49].

The abbreviated name of the subspecies are: par, parva; pyr, pyrenaica; orn, ornata; cat, cartusiana; rup, rupicapra; tat, tatrica; cap, carpatica; bal, balcanica; asi, asiatica; and cau, caucasica.

### Comparing trees obtained with different datasets

To compare the trees obtained with different sets of sequences we performed *BEAST analysis in different combinations of them. Analysis of all nuclear sequences, introns sequenced in this study plus the sequence of a fragment of the SRY promoter, and of the MC1R gene from previous studies, lead to the same topology as in the intron alone analysis (S1 Fig), although the convergence for TreeHeight. Species was poor, with low ESS values (combined EES for posterior probability = 163). A *BEAST analysis of all nuclear markers plus mtDNA sequences did not converge, the ESS values for every parameter in the combined trace file was null. This is likely due to the different signal among nuclear and mtDNA sequences. In addition, the clock rate obtained for the mtDNA relative to the rate of introns was 78.59 (95% HPD interval 49.67–110.64), a disproportionate value even taking into account the higher mtDNA relative to nuclear DNA evolutionary rate. To compare mtDNA and nuclear species tree topologies, we analysed nuclear sequences (introns+SRY+MC1R gene) in *BEAST with the topology constrained to the three mitochondrial main clades. The posterior probabilities of the analysis with or without prior topology were compared using Bayes factors. The log Bayes factor in favour of the nuclear topology over the mtDNA one was 20.16 (Bayes factor = 1.4E20), showing the strong difference between nuclear and mitochondrial topologies.

## Discussion

One of the most used molecules to infer phylogenies and define species is mtDNA [54], however, results in model species in the last years have shown that different components of the genome can show striking differences in the patterns and/or timings of differentiation [55]. Thus, to capture the complexity of the evolutionary history, it is necessary to include genome components with different modes of inheritance. Among the nuclear markers useful for phylogenetic studies of close related species, introns have several desirable features, such as high variability (expected due to nearly neutral evolution) and facility of amplification with primers placed in the adjacent exons [31]. Multilocus phylogenies based on introns have been used lately to study the evolutionary relationships among closely related species and compared to mtDNA observations. We obtained phylogenies of multiple intron loci and compared them to other nuclear loci and to the mitochondrial gene tree using coalescent-based multilocus methods. Discrepancies among nuclear loci can be attributed to ILS, while the conflict between nuclear and mtDNA phylogenies is better explained by introgression among differing lineages in the central area of Europe. Differing patterns of phylogeographic variation allow elucidating the complexity of evolutionary history.

### Diversity of nuclear introns in the genus Rupicapra

The total nucleotide diversity in the genus is 0.1%. Even though the variation across subspecies is clearly partitioned into three groups, the nucleotidic differences among groups are in the order of 0.1–0.2% and there is extensive haplotype sharing. This variation is in the lower range of the intraspecific nucleotide diversity in other mammals [56], including different species of bovids [29], and much lower than the reported pairwise nucleotide distances between close species. Overall, this result contrasts with the high nucleotidic diversity found for mtDNA that reached an overall value of 3.6% and a means of 0.6% within subspecies [19]. With this low diversity, the studied introns have low phylogenetic signal to analyze relationships between chamois populations. In this case, concatenation methods can outperform species tree methods in obtaining the relationships among groups [57]. Our results under concatenation or species tree methods similarly yielded three clear groups, also obtained through traditional AMOVA. The same three groups were recovered from the comparison of microsatellite markers [21] and the MC1R gene [22]. Three main clades concurring in distribution with the nuclear ones except in central Europe, were also referred for mtDNA [18] but, besides the discordant topology regarding the position of *cartusiana* and several individuals from the west Italian Alps, the mtDNA and nuclear markers differ drastically in the depth of differentiation.

The *Rupicapra* root for the concatenated intron tree is 0.0008 substitutions per nucleotide while it is 0.0378 for mtDNA. This huge difference cannot be accounted for by differences in the nuclear and mitochondrial clock, as becomes evident from the comparison of the estimated age of the root in years after calibration. The age of the mtDNA MRCA of *Rupicapra* was estimated to 1.93 MYA (1.56–2.33 CI, 95% HDP) [25]. In contrast, *Rupicapra* introns are remarkably young, 292 ky (95% HDP 241–527 ky), according to data based on concatenation and much more recent following the species tree method. The mean time to coalescence for neutral nuclear markers is 4Ne generations [58], for mtDNA this time is about four times shorter assuming equal effective size in males and females. Therefore, monophyly is expected to occur more rapidly for mitochondrial than for nuclear markers. In addition, a few migrants per generation would prevent monophyly of otherwise isolated populations [59]. This could explain the faint differentiation between populations but not the general low variability of biparental introns that leads to the estimation of short divergence times together with small effective sizes. The low ancestral Ne estimate for the *Rupicapra* genus from our intron dataset (Ne = 7000) can be interpreted as an indication of recent founder events and intense gene flow, and this should have occurred through male dispersal, given the previously determined extremely low diversity of the SRY and microsatellite loci on the Y-chromosome [23]. Agreeing with these observations, small differentiation among chamois species has been reported for the major histocompatibility complex (MHC) class II DBR locus. The proportion of synonymous differences between *R*. *pyrenaica* and *R*. *rupicapra* was low and the two species shared alleles, even more recombination events have probably taken place between alleles of the two species [60]. These observations led to the proposition that the two species could have been in contact during the last glacial maximum when chamois roamed over wide areas in central Europe [60]. Several other studies of nuclear markers showed limited allozyme variability among close chamois populations [61–63]. In addition, it can be noted that the levels of differentiation for microsatellites was low when compared to other mammal populations (see Pérez et al.[21]) and differentiation showed a clear correlation with geographical distance. All these studies of nuclear markers indicated high levels of gene flow among chamois populations and low general variability in the whole genus except for mtDNA.

### Taxonomical implications

The chamois has been classified into one [10], two [12], three [8], six [16], or seven species [17]. Although molecular data did not gave a clear support to the one, two, or three species hypotheses (different markers produced partially discordant phylogenies that alternatively support the different hypotheses), they clearly rejected the six or seven species classifications. The mitochondrial DNA data provide information about phylogeny that is frequently used to diagnose species using the phylogenetic species concept (PSC). Evolutionary Significant Units (ESUs), essentially equivalent to species under the PSC, have been defined as populations of individuals reciprocally monophyletic for mtDNA alleles and differing significantly in the frequency of alleles at nuclear loci [64]. To define different phylogroups as representative of different species, Barker and Bradley [54] proposed that hybridization is restricted to a limited geographic area, and outside the hybrid zones respective phylogroups are defined by unique and concordant statistically supported monophyletic clades based on mitochondrial and nuclear genetic variation. Overall, phylogeographic analysis of mtDNA and nuclear markers allows the definition of three groups of chamois that separate in an east-west pattern and could be thought of as different species assuming introgression in the central area of Europe [19]. Nevertheless, the results presented here on the extremely low differentiation for introns, even in the lower range if one single species is considered, again point to the one species classification.

The issue of the questionable utility of mtDNA for species delineation due to non-neutrality, variation in mutation rate among lineages and introgression has already been raised [55, 65]. From this point of view, our findings highlight that mtDNA phylogroups can be misleading due to introgression. In addition, divergence estimates can be very different from species isolation time if there is female philopatry.

### Integrating molecular variation and the fossil record

In the year 1968, Kurtén wrote that the origin of *Rupicapra* “is a mystery”, what Lovari [66] attributed to the rarity of paleontological remains associated to nature of the terrain where *Rupicapra* lives. For years, the older known fossils of *Rupicapra* were placed in the middle Pleistocene (250–150 Kya) at Caune de l’Arago in the Western Pyrenees [67]. But recently, more ancient fossils were discovered in the Balkans in a biostratigraphic zone that corresponds to between 780 and 750 Kya [27]. Diversification time estimates between mitochondrial lineages based on partial sequences [18–20, 24, 68] or in complete mitochondrial genomes [25] were older, between 1.5 and 2.1 mya. Following the comparison of complete mitochondrial genomes, the MRCA of *Rupicapra* occurred at 1.93 MYA (1.56–2.33 CI, 95%HPD) [25]. This age corresponds to the boundary between the Gelasian and the Calabrian Stages at the Early Pleistocene (following the recent “Formal Subdivisions of the Pleistocene Series/Epoch” of the subcommission on Quaternary Stratigraphy, former Plio-Pleistocen boundary). The biochronological unit known as Villafranchian is considered to represent the earliest stage of the European continental Pleistocene [69] and, interestingly, is characterized by the first occurrence of the Rupicaprini *Gallagoral meneghinii* and *Procamptoceras brivatense* [69]. Although *Procamptoceras* is phenetically close to *Rupicapra*, direct ancestry was ruled out on the basis of cranial morphology (Schaub 1923, cited in [67]). Divergence times estimated from mitochondrial genomes fit well with the proposal of Masini and Lovari [67] that the arrival of chamois or its direct ancestor in Europe could be related to the dispersal events that took place during the Villafranchian. Since then, small populations of *Rupicapra* ancestors possibly remained moving to higher or lower areas, within limited ranges, during interglacial and glacial cycles of Pleistocene, given that the mtDNA clades maintain a conspicuous geographical signature. As said, *Rupicapra* fossils from before the late Pleistocene are scarce. On the contrary, during the Würm age and until the lower Holocene, *Rupicapra* fossils are widespread and were found in low altitude sites through western and eastern Europe [70]. The intron based dating places the common ancestor just before this period of chamois expansion during the Last Glacial Maximum. The dispersal of some few migrant males should have rapidly spread trough the populations of chamois, given the homogeneity of nuclear markers. From the pattern of differentiation of Y-chromosome microsatellites, we have proposed that males dispersed west to east [23] and attending to intron variability it can be argued that male gene flow between proximate populations lasted until the Holocene. The rapid replacement of nuclear genetic variants suggests that at the beginning of male migration, the receptor populations were small and the chamois expansion succeeded from this exchange between genetically distant lineages. Hybridization between distant lineages increases variation that could be subject to adaptive selection [71]. The existence of fixed differences between three lineages for the MC1R gene and the absolute lack of polymorphism within lineage suggest that it was subject to selection. Discordance between the patterns of mitochondrial and nuclear differentiation at a regional scale were detected in populations of the Eastern Alps [72]. There, the mtDNA presented a marked geographical structuring that was not paralleled by variation at 33 allozyme nuclear loci. This discrepancy was attributed to sex-specific dispersal of males and philopatry of the females. To explain the striking differentiation for the maternal genome, the dispersion should have occurred exclusively through males. In fact, direct observation of the dispersal behaviour of marked animals showed a high rate of dispersal in males and more than 90% of philopatric females [73]. The resident females remained at home, in strictly limited areas, since the beginning of Pleistocene, while presumably males wandered largely across Europe, Turkey and the Caucasus during the last glacial period.

It has been shown that time estimates without the use of tip dating, based on ancient DNA sequences form known-age fossils remains, can lead to overestimation of divergence times [74–76]. Therefore, it would be very interesting to sequence *Rupicapra* fossils in the future to check the consistency of molecular dating. In any case, it is clear that timing estimates for mitochondrial or nuclear sequences differ considerably.

Our results highlight that different components of the genome can show conflicting phylogenetic patterns that should be combined to obtain the evolutionary history of organisms. Female philopatry and male dispersal can lead to highly differing phylogenies questioning the general utility of mtDNA in species delimitation. To explain the differing patterns of variation, we should take into account the dynamic nature of the distribution of populations during the extreme glacial oscillations of the Quaternary. Small herds of chamois or their ancestor should have persisted in Europe through glacial oscillations retracting to southern low altitude areas during glacial maxima. At the late Pleistocene, before the last glacial maximum, young male lineages spread through Europe and propelled the subsequent large expansion of chamois populations during the Würm age.

Future studies to understand the evolution of the *Rupicapra* genus should ideally include both the analysis of *Rupicapra* fossils and the analysis of the nuclear genome of the different subspecies (at least *pyrenaica*, *cartusiana*, *ornata* and *rupicapra*) to adjust dating of lineages and disentangle the effects and direction of hybridization events and the extent of gene flow and adaptive selection in the differentiation of chamois.

## Supporting Information

S1 Table Samples used in the study. (DOC)

S2 Table List of the 23 introns and primers used in this study. (DOC)

S3 Table List of GenBank accession numbers for intron sequences. (DOC)

S4 Table List of GenBank accession numbers for SRY promoter, MC1R gene and mtDNA sequences. (DOC)

S5 Table Values of differentiation between groups of populations. (DOC)

S1 Fig Species tree obtained from the analysis of nuclear sequences, 23 introns (15,411 nt), a fragment of the SRY promoter (531 nt) and the MC1R gene (954 nt), in *BEAST. Numbers at the nodes are posterior probabilities.
(PDF)
