# Supplementary material for: Multilocus Intron Trees Reveal Extensive Male-Biased Homogenization of Ancient Populations of Chamois (Rupicapra spp.) across Europe during Late Pleistocene
Source: PLoS One. 2017 Feb 1;12(2):e0170392. doi: 10.1371/journal.pone.0170392 (PMC5287467; doi:10.1371/journal.pone.0170392)
Supplement: S1 Table — (DOC) [file pone.0170392.s001.doc]

| **Subspecies** | **Lab Code** | **Location** | **Sent by** |
| --- | --- | --- | --- |
| *R. pyrenaica parva* | CBWo24 | Cantabrian Mountains, Leitariegos | Game wardens of Principado de Asturias |
| *R. pyrenaica parva* | CBEo12 | Cantabrian Mountains, Piloña | Game wardens of Principado de Asturias |
| *R. pyrenaica pyrenaica* | PYWo15 | Pyrenean Mountain, Benasque | Paloma Barrachina |
| *R. pyrenaica pyrenaica* | PYEo13 | Pyrenean Mountain, Setcases | Game wardens of Gerona |
| *R. pyrenaica ornata* | ANo01 | Apennines, Abruzzo National Park | Luca Rossi |
| *R. rupicapra cartusiana* | CHAv01 | Chartreuse Mountains, Belledonne | Jacques Michallet |
| *R. rupicapra cartusiana* | CHAv04 | Chartreuse Mountains, Belledonne | Jacques Michallet |
| *R. rupicapra rupicapra* | ALWo09 | Alps, Val di Susa | Luca Rossi |
| *R. rupicapra rupicapra* | ALEo03 | Alps,Tarvisio | Luca Rossi |
| *R. rupicapra tatrica* | TAo02 | Tatra Mountains, Tatra National Park | Tomas Skalski |
| *R. rupicapra carpatica* | CPo03 | Carpathian Mountains, Fagaras | Juan Bejar |
| *R. rupicapra balcanica* | BAo16 | Pindos Mountains, Timfi-Vikos | Haritakis Papaioannou |
| *R. rupicapra asiatica* | TUo01 | Kaçkar Mountains, Anatolia | Alvaro Mazón |
| *R. rupicapra caucasica* | CUo05 | Caucasus Mountains, Khevsureti | Jason Badridze |

S1 Table_ Samples sequenced in the study
