# Supplementary material for: Multilocus Intron Trees Reveal Extensive Male-Biased Homogenization of Ancient Populations of Chamois (Rupicapra spp.) across Europe during Late Pleistocene
Source: PLoS One. 2017 Feb 1;12(2):e0170392. doi: 10.1371/journal.pone.0170392 (PMC5287467; doi:10.1371/journal.pone.0170392)
Supplement: S2 Table — (DOC) [file pone.0170392.s002.doc]

| **Gene**  **(intron nº)** | **Description** | **Chr**  **Bos/ovis** | **fragment size ***  **(bp)** | **Forward primer** | **Reverse primer** | **T annealing (ºC)** | **Ref.** |
| --- | --- | --- | --- | --- | --- | --- | --- |
| TRAPPC10 (9) | Trafficking Protein Particle Complex 10 | 1/1 | 631 | ACACCGTGGGCTTGTGGAGC | TCCACACAGATAGCCCAGGG | 62º | Hailer et al. 2012 |
| CLCA1 (12) | Chloride Channel Accessory 1 | 3/1 | 983 | **GGCAAATTCCCTAGCCCTATG** | **GCCCACACTTTTRCACTGTATC** | 60º | Igea et al. 2010 |
| LRGUK (14) | Leucine-Rich Repeats And Guanylate Kinase Domain Containing | 4/4 | 763 | ATCCAGGRTACTTTGATGCAG | CTSAGCTTTTGGTAGGCAAC | 62º | Hailer et al. 2012 |
| SEL1L3 (20) | Sel-1 Suppressor Of Lin-12-Like 3 (C. Elegans) | 6/6 | 1096 | TTCTTGGAAATTGACCCAAC | GGCTGAAGGACTCCTCATTG | 60º | Hailer et al. 2012 |
| COPE (6) | Coatomer Protein Complex, Subunit Epsilon | 7/5 | 1079 | **AAGATGCAGGACCAGGACGA** | **GCCATCTCCTGGAAGATGTAGT** | 60º | Igea et al. 2010 |
| ABCA1 (49) | ATP-Binding Cassette, Sub-Family A (ABC1), Member 1 | 8/2 | 724 | TCCTTTCCCAGAGCAAAAAG | TGGTCCTTGGCAAAGTTTAC | 60º | Hailer et al. 2012 |
| HDAC2 (13) | Histone Deacetylase 2 | 9/8 | 731 | GACAAACCAGAACACTCCAG | GGTGCATGAGGCAACATGCGT | 60º | Hassanin et al. 2013 |
| PABPN1 (2) | Poly(A) Binding Protein, Nuclear 1 | 10/7 | 766 | GAAGCAGATGAATATGAGTCCACC | CTTCTCCTCAATGGACATGAT | 60º | Hassanin et al. 2013 |
| SPTBN1 (31) | Spectrin, Beta, Non-Erythrocytic 1 | 11/3 | 746 | AGACTGAGTCCCAGCAGCAG | CCATTCTGGGAAACTTGSTC | 62º | Hailer et al. 2012 |
| ATP12A (14) | ATPase, H+/K+ Transporting, Nongastric, Alpha Polypeptide | 12/10 | 744 | CCAAGTGCCGGAGYGCAGGG | CTTGGCAATRGCTTTGGCTG | 65º | Hailer et al. 2012 |
| GAD2 (1) | Glutamate Decarboxylase 2 (Pancreatic Islets And Brain, 65kDa) | 13/13 | 752 | **GATCCCGAGAACCCCGGCACAG** | TCCGATGCCGCCCGTGAACTTCT | 62º | Igea et al. 2010 |
| AZIN1 (8) | Antizyme Inhibitor 1 | 14/9 | 666 | CATTGGTGGRGGCTTCACAG | AGACATCCAACAAAGGGCTG | 60º | Hailer et al. 2012 |
| LYVE1 (5) | Lymphatic Vessel Endothelial Hyaluronan Receptor 1 | 15/15 | 653 | **GCAGTYTGCTACRTCAAAAGGTG** | **GGGTTGCTATCATCGGCCTTCT** | 60º | Igea et al. 2010 |
| PTGS2 (3) | Prostaglandin-Endoperoxide Synthase 2 (Prostaglandin G/H Synthase And Cyclooxygenase) | 16/12 | 845 | **ACCCACTTCAARGGAGTCTGGA** | **GACAGGTTAGAAAAGGCTTCCCA** | 59º | Igea et al. 2010 |
| FGB (8) | Fibrinogen Beta Chain | 17/17 | 739 | **CCACAACAGCATGTTCTTCAGCA** | **GTATCTGCCATTGGGATTGGCT** | 60º | Igea et al. 2010 |
| GGA3 (4) | Golgi-Associated, Gamma Adaptin Ear Containing, ARF Binding Protein 3 | 19/11 | 646 | GGGGAAGTTCCGSTTTTTG | TTTCTCAGACACCCTGTCCC | 60º | Hailer et al. 2012 |
| PNN (1) | Pinin, Desmosome Associated Protein | 21/18 | 808 | **GCAGCTAGAAAAGGCCAAAG** | **AACTACCACGTCCTCTACCTC** | 60º | Igea et al. 2010 |
| SCN5A (26) | Sodium Channel, Voltage Gated, Type V Alpha Subunit | 22/19 | 712 | GTTCATCGGTGTCATCATTG | TGTCATGAAGATGTCCTGGC | 62º | Hailer et al. 2012 |
| RIOK3 (6) | RIO Kinase 3 | 24/23 | 744 | GCCTACTCAGAAGAACGTCGAAG | ACGTGTCTTAGGATCAACTG | 60º | Hassanin et al. |
| CARHSP1 (2) | Calcium Regulated Heat Stable Protein 1, 24kDa | 25/24 | 769 | ACTCGCCGCACCAGGACCTWCT | GTGATGAAGCCGTGGCCCTTGGA | 62º | Igea et al. 2010 |
| TUFM (9) | Tu Translation Elongation Factor, Mitochondrial | 25/24 | 885 | CTGACTTGGGACATGGCCTGTCG | ACGCTGGCCTTTCTCYAAGATCAT | 60º | Hassanin et al. 2013 |
| ZFYVE27 (6) | Zinc Finger, FYVE Domain Containing 27 | 26/22 | 734 | GAATGTGGAGTTCTTCCGAG | GGGTTCATCCGCCGCTGCAGA | 60º | Hassanin et al. 2013 |
| KLC2 (11) | Kinesin Light Chain 2 | 29/21 | 521 | **CAGCTGGTACAAGGCCTGTAAAGT** | **TGTGYGCGGCTTCCAGYTT** | 60º | Igea et al. 2010 |

S2 Table._ List of the 23 introns used in this study. Intron number and PCR fragment size correspond to *Bos taurus*. In bold: primers designed “de novo” for this work
