# Supplementary material for: Multilocus Intron Trees Reveal Extensive Male-Biased Homogenization of Ancient Populations of Chamois (Rupicapra spp.) across Europe during Late Pleistocene
Source: PLoS One. 2017 Feb 1;12(2):e0170392. doi: 10.1371/journal.pone.0170392 (PMC5287467; doi:10.1371/journal.pone.0170392)
Supplement: S3 Table — (DOC) [file pone.0170392.s003.doc]

S3. Table_ List of GenBank accession numbers for intron sequences

| **Gene**  **(intron nº)** | **GenBank accession numbers** |
| --- | --- |
| TRAPPC10 (9) | KU343092 - KU343105 |
| CLCA1 (12) | KU342868 - KU342881 |
| LRGUK (14) | KU342966 - KU342979 |
| SEL1L3 (20) | KU343064 - KU343077 |
| COPE (6) | KU342882 - KU342895 |
| ABCA1 (49) | KU342812 - KU342825 |
| HDAC2 (13) | KU342938 - KU342951 |
| PABPN1 (2) | KU342994 - KU343007 |
| SPTBN1 (31) | KU343078 - KU343091 |
| ATP12A (14) | KU342826 - KU342839 |
| GAD2 (1) | KU342910 - KU342923 |
| AZIN1 (8) | KU342840 - KU342853 |
| LYVE1 (5) | KU342980 - KU342993 |
| PTGS2 (3) | KU343022 - KU343035 |
| FGB (8) | KU342896 - KU342909 |
| GGA3 (4) | KU342924 - KU342937 |
| PNN (1) | KU343008 - KU343021 |
| SCN5A (26) | KU343050 - KU343063 |
| RIOK3 (6) | KU343036 - KU343049 |
| CARHSP1 (2) | KU342854 - KU342867 |
| TUFM (9) | KU343106 - KU343119 |
| ZFYVE27 (6) | KU343120 - KU343133 |
| KLC2 (11) | KU342952 - KU342965 |
