# Supplementary material for: Multilocus Intron Trees Reveal Extensive Male-Biased Homogenization of Ancient Populations of Chamois (Rupicapra spp.) across Europe during Late Pleistocene
Source: PLoS One. 2017 Feb 1;12(2):e0170392. doi: 10.1371/journal.pone.0170392 (PMC5287467; doi:10.1371/journal.pone.0170392)
Supplement: S4 Table — (DOC) [file pone.0170392.s004.doc]

S4 Table._ Samples and GenBank accession numbers for sequences of the SRY promoter, the MC1R gene and the mtDNA regions used

| **Subspecies** | **Lab Code** | **Location** | **SRY promoter** | **MC1R gene** | **mtDNA regions** | | | | |
| --- | --- | --- | --- | --- | --- | --- | --- | --- | --- |
|  |  |  |  |  | cytb | ND1 | 12S | tRNApro | CR |
| *R. pyrenaica parva* | CBWo21 | Cantabrian Mountains, Somiedo |  |  | EU836161 | GU951809 | GU951833 | GU951830 | GU951843 |
| *R. pyrenaica parva* | CBWo02 | Cantabrian Mountains, Somiedo | JN547785 | JX868509 |  |  |  |  |  |
| *R. pyrenaica parva* | CBEo17 | Cantabrian Mountains, Ponga |  | JX868509 | EU836163 | GU951810 | GU951833 | GU951830 | GU951849 |
| *R. pyrenaica parva* | CBEo21 | Cantabrian Mountains, Ponga | JN547785 |  |  |  |  |  |  |
| *R. pyrenaica pyrenaica* | PYWo15 | Pyrenean Mountain, Benasque | JN547785 | JX868509 | EU836160 | GU951812 | GU951833 | GU951830 | GU951856 |
| *R. pyrenaica pyrenaica* | PYEo13 | Pyrenean Mountain, Setcases |  | JX868509 | EU836160 | GU951814 | GU951833 | GU951830 | GU951860 |
| *R. pyrenaica pyrenaica* | PYEo04 | Pyrenean Mountain, P.N. Aigues Tortes | JN547785 |  |  |  |  |  |  |
| *R. pyrenaica ornata* | ANo01 | Apennines, P.N. Abruzzo | - | JX868510 | EU836164 | GU951815 | GU951834 | GU951831 | GU951864 |
| *R. pyrenaica ornata* | ANv830 | Apennines, P.N. Abruzzo | JN547785 |  |  |  |  |  |  |
| *R. rupicapra cartusiana* | CHAv01 | Chartreuse Mountains |  | JX868511 | EU836158 | GU951816 | GU951834 | GU951831 | GU951865 |
| *R. rupicapra cartusiana* | CHAv04 | Chartreuse Mountains | JN547786 | JX868511 | EU836158 | GU951816 | GU951834 | GU951831 | GU951865 |
| *R. rupicapra cartusiana* | CHAv06 | Chartreuse Mountains | JN547786 |  |  |  |  |  |  |
| *R. rupicapra rupicapra* | ALWo09 | Alps, Val di Susa | JN547786 |  | EU836160 | GU951818 | GU951833 | GU951830 | GU951873 |
| *R. rupicapra rupicapra* | ALWo03 | Alps, Val di Susa |  | JX868511 |  |  |  |  |  |
| *R. rupicapra rupicapra* | ALEo03 | Alps,Tarvisio | JN547786 |  | EU836151 | GU951817 | GU951837 | GU951832 | GU951886 |
| *R. rupicapra rupicapra* | ALEo05 | Alps,Tarvisio |  | JX868511 |  |  |  |  |  |
| *R. rupicapra tatrica* | TAo02 | Tatra Mountains, P.N.Tatra | JN547786 |  | EU836156 | GU951826 | GU951837 | GU951832 | GU951899 |
| *R. rupicapra tatrica* | TAv102 | Low Tatra |  | JX868511 |  |  |  |  |  |
| *R. rupicapra carpatica* | CPo03 | Carpathian Mountains, Fagaras | JN547786 |  | EU836156 | GU951826 | GU951837 | GU951832 | GU951904 |
| *R. rupicapra carpatica* | CPo05 | Carpathian Mountains, Azuga |  | JX868511 |  |  |  |  |  |
| *R. rupicapra balcanica* | BAo16 | Balkan Mountains, Timfi-Vikos | JN547786 | JX868511 | EU836156 | GU951825 | GU951837 | GU951832 | GU951895 |
| *R. rupicapra asiatica* | TUo01 | Anatolia. Kackar Mountains | JN547786 | JX868511 | EU836156 | GU951817 | GU951837 | GU951832 | GU951912 |
| *R. rupicapra caucasica* | CUo05 | Caucasus Mountains, Khevsureti |  | JX868511 |  |  |  |  |  |
| *R. rupicapra caucasica* | CUo02 | Caucasus  Mountains,  North Ossetia | JN547786 |  | EU836156 | GU951817 | GU951837 | GU951832 | GU951913 |
