# Supplementary material for: Multilocus Intron Trees Reveal Extensive Male-Biased Homogenization of Ancient Populations of Chamois (Rupicapra spp.) across Europe during Late Pleistocene
Source: PLoS One. 2017 Feb 1;12(2):e0170392. doi: 10.1371/journal.pone.0170392 (PMC5287467; doi:10.1371/journal.pone.0170392)
Supplement: S5 Table — (DOC) [file pone.0170392.s005.doc]

S5 Table._ Values of differentiation between groups of populations. a) Pairwise Fst values (below) and corresponding P values (above). b) Pairwise PhiST (below) and corresponding P values (above)

a)

| **Group** | **iberica** | **ornata** | **rupicapra** |
| --- | --- | --- | --- |
| **iberica** | - | 0.001 | 0.000 |
| **ornata** | 0.785 | - | 0.000 |
| **rupicapra** | 0.634 | 0.682 | - |

b)

| **Group** | **iberica** | **ornata** | **rupicapra** |
| --- | --- | --- | --- |
| **iberica** | - | 0.02444 | 0.00000 |
| **ornata** | 0.82291 | - | 0.00293 |
| **rupicapra** | 0.77834 | 0.82539 | - |
