# Supplementary figures and images for: Multilocus Intron Trees Reveal Extensive Male-Biased Homogenization of Ancient Populations of Chamois (Rupicapra spp.) across Europe during Late Pleistocene
Source: PLoS One. 2017 Feb 1;12(2):e0170392. doi: 10.1371/journal.pone.0170392 (PMC5287467; doi:10.1371/journal.pone.0170392)

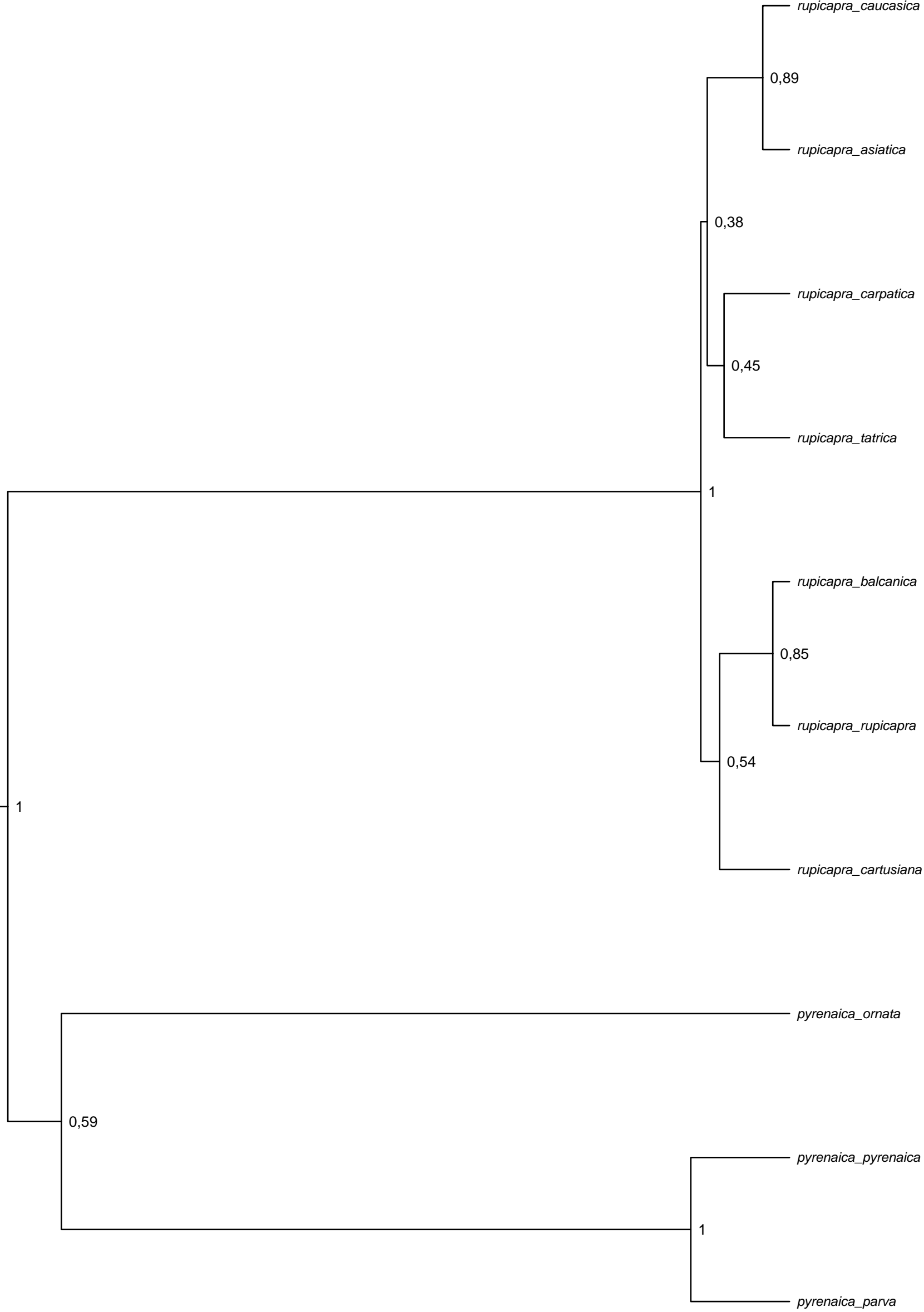

Supplement: S1 Fig — Numbers at the nodes are posterior probabilities. (PDF) [file pone.0170392.s006.pdf]
